# FedMann: A Manually Coordinated Federated Learning Framework Applied to Multiple Myeloma—A Feasibility Study

**DOI:** 10.3390/jcm15218418

**Published:** 2026-10-29

**Authors:** Stuart W. Sommers, Chunlei Zheng, June K. Corrigan, John M. Culnan, Geethika Bodanapu, Grace M. Ferri, Maya Abdallah, Britney Bell, Meghana Yellanki, Danne C. Elbers, Nhan V. Do, Mary T. Brophy, Nikhil C. Munshi, Camille V. Edwards, Nathanael R. Fillmore

**Affiliations:** 1 VA Boston Healthcare System, Boston, MA 02301, USA; stuart.sommers@va.gov (S.W.S.); nhan.do@va.gov (N.V.D.); camille_edwards@dfci.harvard.edu (C.V.E.); 2 Chobanian & Avedisian School of Medicine, Boston University, Boston, MA 02118, USA; 3 Section of Hematology and Medical Oncology, Boston Medical Center, Boston, MA 02118, USA; 4 Accenture Federal Services, Arlington, VA 22203, USA; 5 Department of Medicine, Harvard Medical School, Boston, MA 02115, USA; 6 The Jerome Lipper Multiple Myeloma Center, Dana-Farber Cancer Institute, Boston, MA 02215, USA

**Keywords:** federated learning, multiple myeloma, electronic health records, multi-institutional collaboration, clinical informatics infrastructure

## Abstract

**Background/Objectives**: Federated learning (FL) enables multi-institutional model development in oncology without sharing patient-level data, but automated FL infrastructures typically require dedicated servers, firewall modifications, and substantial IT support to deploy. These requirements may be prohibitive for smaller institutions or consortia with limited IT resources or stringent information security requirements. We developed FedMann, a manually coordinated FL framework, and demonstrated its feasibility by applying it to prediction of suboptimal multiple myeloma (MM) treatment across two clinical sites with substantially different patient populations. **Methods**: This retrospective cohort study used electronic health record data from patients newly diagnosed with MM at the Veterans Affairs (VA) Healthcare System or Boston Medical Center (BMC) between 2012 and 2022. Demographic, clinical, and geographic predictors were used to predict non-receipt of guideline-concordant primary therapy, defined as not receiving a frontline triplet or quadruplet regimen or autologous stem cell transplant when eligible. FedMann was implemented as a four-stage cycle of local training, parameter sharing through an access-controlled repository, sample-size-weighted parameter averaging at a coordinating site, and federated model redistribution. Models were evaluated on site-specific held-out test sets using area under the receiver operating characteristic curve (AUC) with bootstrap-resampled 95% confidence intervals. **Results**: VA and BMC contributed 4887 and 261 cases, respectively, with substantial demographic differences across all measured characteristics including sex distribution (96.3% male at VA vs. 58.6% at BMC; *p* < 0.001). The BMC-only model achieved an AUC of 0.70 (95% CI, 0.56–0.81) on the BMC test set. The federated model achieved 0.68 (95% CI, 0.55–0.80) on the BMC test set and 0.70 (95% CI, 0.67–0.72) on the VA test set. **Conclusions**: A manually coordinated FL framework can support multi-institutional model fitting in oncology without exchanging patient-level data and without requiring dedicated FL infrastructure. In a two-site MM application, performance was comparable between the federated model and each site’s own local model, and the VA only model showed no apparent loss of discrimination when externally validated at BMC, with no statistically significant differences in AUC on the BMC test set. Although absolute AUCs were modest, reflecting limited cohort size and a small predictor set, this application demonstrates the feasibility of the framework as an alternative when automated FL deployment is impractical.

## 1. Introduction

Multiple myeloma (MM) is a malignant plasma cell disorder accounting for approximately 32,000 new cases and 12,000 deaths annually in the United States [1]. Despite advances in therapy that have substantially extended survival, disparities in receipt of guideline-concordant primary therapy persist. Patients with lower socioeconomic status, inadequate insurance coverage, or greater geographic distance from specialized centers are less likely to receive guideline-recommended frontline regimens, including triplet or quadruplet drug combinations and autologous stem cell transplantation when eligible [2,3]. These gaps in care contribute to worse survival and represent a tractable target for intervention.

Identifying patients at risk of not receiving guideline-concordant primary therapy could enable targeted outreach, such as social work engagement or patient navigation, before treatment decisions are finalized. Predictive models for this purpose differ from established prognostic tools in MM, which exclude social factors and focus on survival rather than treatment receipt [4,5]. Developing a generalizable model for treatment receipt requires data from diverse populations [6,7], since the social and structural determinants that drive disparities vary across health systems and geographic regions [3]. However, multi-institutional model development is constrained by patient privacy regulations and the practical burdens of data sharing, including data use agreements, harmonized data dictionaries, and shared compute infrastructure [8].

Federated learning (FL) offers an alternative in which each institution trains a local model on its own data and shares only model parameters, rather than patient-level records, with a coordinating site that aggregates them into a combined model [9,10,11,12]. This approach preserves data custody at each site while enabling joint model development. Most published FL applications in healthcare have used automated infrastructures that require dedicated servers, firewall modifications, and substantial IT support to deploy [9]. These requirements can be prohibitive for smaller institutions or consortia with limited IT resources or stringent information security requirements, and they have limited the breadth of sites that can participate in collaborative modeling. Manual coordination, in which sites exchange model parameters through a shared repository under human oversight, is a less infrastructure-intensive alternative that has received less methodological attention.

Our objective was to develop and evaluate the feasibility of a manually coordinated FL framework, which we call FedMann, for fitting models across clinical sites with heterogeneous patient populations. We describe the framework, apply it to the prediction of non-receipt of guideline concordant primary therapy using electronic health record (EHR) data from two healthcare systems, and report its performance relative to site-specific and externally validated models.

## 2. Materials and Methods

### 2.1. Data Sources and Cohorts

This retrospective cohort study used EHR data from patients newly diagnosed with MM in the nationwide Veterans Affairs (VA) healthcare system or at Boston Medical Center (BMC) between 2012 and 2022. Tabular demographic, clinical, and geographic data were extracted from each site’s EHR using previously validated algorithms [13,14]. Categorical variables were used to compare demographic distributions between sites with chi-square tests of independence. The study was approved by the VA Boston Research and Development Committee and the Boston University Medical Center Institutional Review Board as an exempt study prior to data collection and analysis, with a waiver of informed consent due to the use of existing data, per the Common Rule. A formal study protocol was not prepared prior to analysis. The study followed the Transparent Reporting of a multivariable prediction model for Individual Prognosis Or Diagnosis + Artificial Intelligence (TRIPOD+AI) reporting guidelines (Collins 2024; Table S1) [15]. The study was not prospectively registered in a clinical trial registry. Newly diagnosed MM was operationalized as an incident diagnosis recorded in the VA Central Cancer Registry or, at BMC, in the EHR between 2012 and 2022. Patients were included only when both the incident diagnosis and first-line induction therapy were documented within the same system within 90 days of diagnosis; patients diagnosed at a participating site or whose induction therapy was delivered elsewhere were not captured. Missing covariates were coded as a separate category rather than used to exclude patients from the analytic cohort. Because the VA and BMC datasets could not be linked at the patient level, duplicate detection across the two databases was not performed. Cross database overlap is expected to be minimal given the aforementioned eligibility window and the largely distinct populations served by a national integrated system and a single safety-net hospital. That said, overlap cannot be fully excluded. At BMC, candidate patients were initially identified using diagnosis codes (approximately 800 records). Duplicate records within this extraction were removed manually, and diagnosis was confirmed by clinician review of the EHR.

### 2.2. Outcome and Predictors

The outcome was a binary indicator of non-receipt of guideline concordant primary MM therapy, defined as not receiving a frontline triplet or quadruplet regimen or autologous stem cell transplant within 90 days of MM diagnosis. A triplet, quadruplet, or ASCT-based approach was considered guideline-concordant primary therapy because each falls within the National Comprehensive Cancer Network (NCCN) treatment algorithm for newly diagnosed MM. Rather than adjudicating which regimen was optimal for a given patient, a determination that depends on individual fitness, frailty, comorbidity, and cytogenetic risk not reliably captured in administrative data, we used receipt of any of these regimens as a marker of guideline-concordant primary therapy because wanted the key distinction to be treated versus untreated. Included therapies were bortezomib, belantamab mafodotin-blmf, bendamustine, carfilzomib, carmustine, cyclophosphamide, cyclophosphamide/mannitol, daratumumab, daratumumab/hyaluronidase-fihj, doxorubicin, doxorubicin/sodium chloride, elotuzumab, etoposide, idecabtagene vicleucel, isatuximab-irfc, ixazomib, lenalidomide, melphalan, panobinostat, pomalidomide, selinexor, thalidomide, and vincristine. Corticosteroid use was not directly captured and was assumed present when a patient received two other agents forming a valid triplet. Valid unique triplet and quadruplet regiments were adjudicated by a hematologist oncologist (study coauthor C.V.E.). For the VA, induction therapy start dates were extracted from structured pharmacy records in the Corporate Data Warehouse, whereas, at BMC therapy dates were derived from a structured dataset extracted from the EHR, supplemented by review of oncology treatment notes where needed. The transplant component of the outcome reflected documented receipt of ASCT rather than an independently adjudicated eligibility determination, i.e., no age, comorbidity, or insurance-based eligibility algorithm was applied. Treatment receipt was abstracted directly from clinical documentation and pharmacy records without intervention during model development or evaluation; no treatments were modified or excluded based on model performance. This outcome definition was held constant throughout the 2012–2022 study period.

Eight predictors were used: age group, sex, race/ethnicity, smoking status, R-ISS stage, Charlson Comorbidity Index (CCI), distance from the hospital, and diagnosis year. These variables were chosen based on literature and availability across sites [3]. More explicitly, the VA did not require insurance and did not collect preferred language data, and BMC did not have income or ADI info accessible, so we had to exclude those four variables from modeling. Furthermore, a cytogenetic factor extraction algorithm failed, thus, cytogenetic data were also excluded from the included predictors to avoid systematic error.

### 2.3. Data Preprocessing

Each site preprocessed its data locally. Categorical predictors were converted to one-hot encoded vectors. Diagnosis year, the only continuous predictor, was centered and scaled using a fixed anchor common to both sites (year minus 2017, divided by five) rather than site-specific statistics, so that its coefficient has the same meaning at each site and remained coherent under parameter averaging. Missing values were imputed using a chained equations approach analogous to Multiple Imputation by Chained Equations (van Buuren 2011) [16], implemented natively in Python v3.11.7 using scikit-learn v1.4.1 (multinomial logistic regression for categorical predictors, linear regression for continuous predictors); A single completed dataset was generated for each site using training data only. The test set was imputed using training-derived reference values only so that no information from held-out data could influence how any value was imputed. Category values were also validated against a fixed set of permissible categories before imputation to identify data-entry inconsistencies. Data were randomly split into training (60%) and test (40%) sets using a fixed random seed to ensure reproducibility. The relatively large test fraction was chosen to obtain stable held-out performance estimates given the limited size of the BMC cohort.

### 2.4. Sample Size Justification

This retrospective cohort study used all available patients meeting inclusion criteria at each participating site during the study period (2012–2022). Rather than a fixed sample size calculation, the analysis was designed to accommodate the actual sample sizes available. The relatively large test set fraction (40%) was deliberately chosen to obtain stable held-out performance estimates given the limited BMC cohort size, prioritizing external validity over training set size. Given the modest number of predictors (8) and the binary outcome, the BMC training set (approximately 157 samples) provided adequate support for logistic regression estimation. This feasibility study was designed to demonstrate the technical feasibility of the FedMann framework rather than to achieve a target performance threshold, and the modest cohort sizes are appropriate for that objective. After the one-hot encoding, the seven categorical predictors and continuous diagnosis year correspond to 28 model parameters plus an intercept (29 total). At BMC, this yielded an events-per-parameter ratio below commonly recommended thresholds for stable logistic regression estimation [17]. L2 regularization (weight decay coefficient 0.001) was applied during training to mitigate instability from this limited events-per-parameter ratio. This constraint, together with the feasibility objective of this study, is discussed further as a limitation below.

### 2.5. FedMann Framework

**Figure 1:**
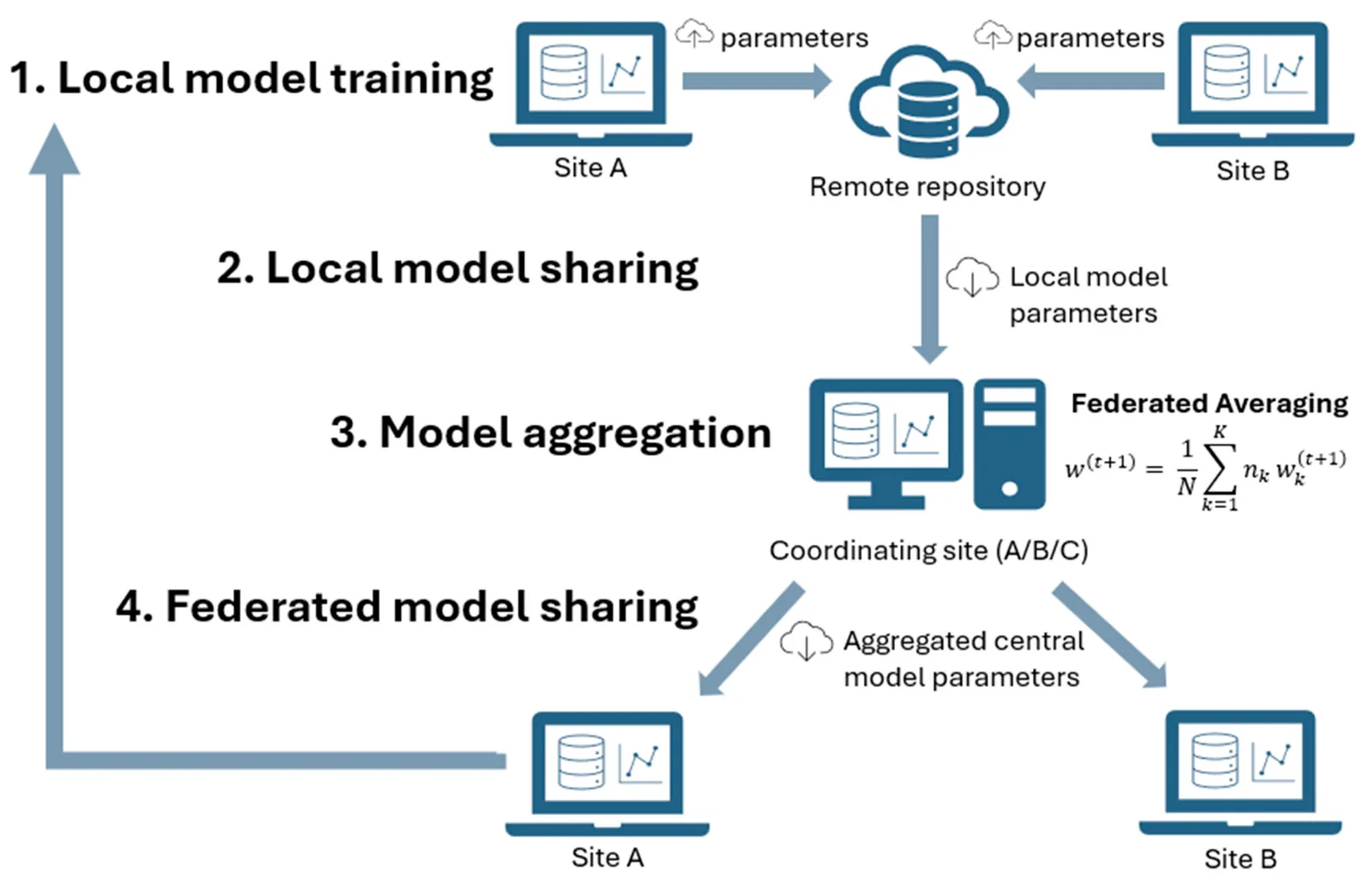
FedMann Framework. Each round of the framework consists of four stages: (1) local model training at each participating site; (2) local model sharing, in which sites upload trained model parameters to a remote repository; (3) model aggregation by sample-size-weighted averaging at a coordinating site; and (4) federated model sharing, in which the aggregated parameters are returned to participating sites for use in the next round. The coordinating site may be one of the participating sites or a separate site. In the aggregation equation, *w*^(*t*+1)^ is the aggregated parameter tensor at round *t* + 1, *K* is the number of sites, *n_k_* is the local training sample size at site *k*, *N* is the total training sample size across sites, and *w_k_*^(*t*+1)^ is the parameter tensor from site *k* at round *t* + 1.

FedMann is a manually coordinated FL framework applicable to any model class for which weighted averaging of model parameters is meaningful, including generalized linear models and shallow neural networks. The framework was applied here to logistic regression. A complete training round consists of four stages (Figure 1): (1) local model training, in which each site independently trains a model on its own data; (2) local model sharing, in which each site uploads its trained model parameters, serialized as PyTorch v2.2.0 parameter objects containing weights and biases, together with its local training sample count, to an access-controlled shared repository; (3) parameter aggregation, in which a designated coordinating site computes a sample-size-weighted average of the parameter tensors; and (4) federated model sharing, in which the aggregated parameters are distributed back to participating sites for use in the next round.

At round *t*, parameters were aggregated using sample-size weighting:

$$w^{\left(t+1\right)}=\;\frac{1}{N}\sum_{k=1}^{K}n_{k}w_{k}^{\left(t+1\right)}$$

where *K* is the number of sites, *n_k_* is site *k*’s local training sample size, *N* is the total training sample size across sites, and *w_k_* is site *k*’s parameter tensor set [18]. Subsequent rounds were initialized from the previous round’s federated model. The cycle continued until performance did not improve for at least two consecutive rounds. Model architecture, parameter naming, and tensor ordering were standardized across sites; before aggregation, scripts verified that parameter keysets matched exactly to prevent misalignment during aggregation.

The framework maintained separate model directories for local models (never shared), shared local models (uploaded for aggregation), and federated models, ensuring clear separation between private and shared model components and facilitating model versioning and reproducibility. Sites retained full custody of patient-level data; only model parameters and aggregate sample counts crossed site boundaries. Local evaluation outputs, including bootstrap-based uncertainty estimates, were written to local logs and not shared. Parameters were exchanged via git v2.51.0.windows.2 using a private repository; access to data required two-factor authentication under organizational access controls, and commits were reviewed before pushing to prevent inadvertent inclusion of patient-level data. Additional security properties, including end-to-end integrity verification (such as checksums or signatures), formal audit logs of each transfer, and institution-specific encryption and access policies, were supported by the surrounding repository and governance workflow rather than enforced within FedMann itself. As a sensitivity analysis, aggregation was repeated using equal weights across sites (1/K per site) rather than sample-size weighting to assess whether the smaller site’s contribution was dominated by the larger site under the default scheme.

### 2.6. Application to Logistic Regression

We applied FedMann to logistic regression as the model class. Logistic regression was implemented in PyTorch v2.2.0 as a single-layer neural network (a single linear layer with sigmoid activation), trained with binary cross-entropy loss and the Adam optimizer. This gradient-based implementation, rather than a conventional solver such as L-BFGS, was required to support iterative weight updates across federated rounds. At each site, processed datasets were loaded into a PyTorch DataLoader for mini-batch processing (batch size = 64, learning rate = 0.01, ten epochs per round), with shuffling enabled across mini-batches at each epoch. Hyperparameters (learning rate, number of epochs, and batch size) were fixed at commonly used defaults rather than selected via a validation procedure. This was again necessitated by the small BMC sample size that discouraged including a tuning split. To confirm that the gradient-based implementation produced results comparable to a conventional solver in a non-federated setting, we compared its performance with logistic regression fit using scikit-learn’s L-BFGS solver (capped at ten iterations to match the number of training epochs).

Class imbalance was not explicitly addressed through sampling or weighting methods, as the outcome prevalence (approximately 25% non-receipt of guideline-concordant primary therapy at both sites) did not constitute severe imbalance. Model performance was evaluated using AUC, which is relatively insensitive to class imbalance. No threshold adjustment or cost-sensitive learning approaches were applied.

The model outputs predicted probabilities in the range [0, 1], representing the estimated probability that a patient would receive guideline-concordant primary MM therapy. No classification threshold was established; probabilistic outputs were used directly for evaluation

### 2.7. Model Evaluation

We defined a local model as a model trained at a single site using only that site’s own data, with no cross-site parameter sharing or aggregation at any point. We defined a federated model as the model produced immediately by sample-size weighted aggregation (FedAvg) of the parameter sets uploaded by all participating sites at the end of a round; this was the model redistributed to sites for use in the next round of local training. We defined a locally fine-tuned model as a model that began training from a federated model’s parameters and then continued training on a single site’s own data, without any further aggregation. Models were evaluated independently at each site using site-specific held-out test sets that were not used during training. Three models were evaluated on each site’s test set: (1) the site’s own model prior to that round’s aggregation (a local model in Round 1 or a locally fine-tuned model in subsequent rounds), representing baseline performance using only local data; (2) the federated model, representing the collaborative learning outcome; and (3) the other site’s own local model, prior to aggregation, therefore representing external validation performance. This design allowed comparison of local-only training against FL performance and assessment of cross-site generalization. All models were evaluated using the same predictors and identical test sets. Performance metrics were the area under the receiver operating characteristic curve (AUC) and accuracy, with bootstrap resampling (n = 2000 replicates) used to estimate 95% confidence intervals. In addition to evaluating the final federated model, we evaluated performance after each round of FedAvg and local re-training to characterize how test-set discrimination changed across steps. We defined a “local model” as one trained at one site without cross-site training or parameter aggregation. We defined a “federated model” or “federated learning” performance as performance of any model after one or more round of FedAvg. Test set performance was used only to characterize discrimination across rounds and to inform when to stop training; it did not contribute to model parameter estimation at any stage.

This study addresses treatment outcome disparities, which motivated the selection of a diverse predictor set and the deliberate inclusion of two healthcare systems with substantially different demographic populations. We evaluated model performance separately by site to assess cross-site generalizability of the federated model across these heterogenous populations, rather than to establish formal algorithmic fairness or equitable performance. This application did not employ formal fairness constraint methods or post hoc fairness interventions.

In addition to AUC and accuracy, we assessed calibration (calibration intercept and slope—estimated via logistic recalibration in statsmodels v0.14.1, calibration curves, and Brier score), and reported sensitivity, specificity, positive predictive value, and negative predictive value at both a fixed probability threshold of 0.5 and a training set derived threshold that maximized Youden’s J statistic. Thresholds were never derived from test set data. We also compared AUCs between the federated local, and externally validated models on the same test set using a paired bootstrap procedure, in which the same resampled patients were used to score each model in every replicate, yielding a 95% confidence interval for the difference in AUC between models.

### 2.8. Data Governance and Security

FedMann is not a comprehensive privacy-preserving solution and does not independently provide formal differential-privacy or cryptographic guarantees. Only logistic regression model parameters were shared via git. Repository activity was traceable via ordinary git commit history. Transit encryption was provided by git over HTTPS/SSH, while at-rest encryption was managed by the underlying platform. Access to data required two-factor authentication under organizational access controls, and commits were reviewed before pushing to prevent inadvertent inclusion of patient-level data. Git is only one possible implementation environment and not an integral component of these methods. FedMann’s own code never calls a git API; it only reads and writes local model directories that a human can then move using git.

## 3. Results

### 3.1. Cohort Characteristics

The VA and BMC contributed 4887 and 261 cases, respectively. Demographic differences between sites were substantial and statistically significant across all predictors (*p* < 0.001; Table 1). VA patients were predominantly male (96.3% vs. 58.6% at BMC), more likely to be current smokers (32.3% vs. 8.8%), more likely to live farther from the hospital (22.5% lived more than 125 km away, vs. 0.8% at BMC), and more likely to be non-Hispanic White (59.7% vs. 22.2%). Despite these differences, the rate of not receiving guideline-concordant primary therapy was similar between sites: 28.1% at VA and 23.0% at BMC (difference 5.1 percentage points; *p* = 0.085).

### 3.2. Baseline Performance Without Federated Learning

On the BMC test set, the local BMC-only logistic regression model achieved an AUC of 0.70 (95% CI: 0.56–0.81). External validation of the VA-only model on the BMC test set achieved an AUC of 0.68 (95% CI: 0.54–0.80). The VA-only model evaluated on its own VA test set achieved an AUC of 0.70 (95% CI: 0.67–0.72). Our PyTorch implementation produced a VA test set AUC of 0.70), compared with 0.70 for logistic regression fit using scikit-learn’s standard solver (Table 2).

### 3.3. Model Performance Using Federated Learning

Three federated rounds were conducted. The federated model’s AUC on BMC test set did not improve after Round 1, meeting the stopping criterion of no improvement for two consecutive rounds. After the first round, in which each site’s local model was trained independently and the parameters were then aggregated, the federated model achieved an AUC of 0.689 (95% CI: 0.55–0.80; Table 3) on the BMC test set. This was comparable to the BMC local model (0.70) and to the externally validated VA model (0.68). In the second and third rounds, training continued at each site starting from the previous round’s aggregated model, followed by re-aggregation. The federated models from the second and third rounds maintained AUCs of 0.68 (95% CI: 0.54–0.80) and 0.68 (95% CI: 0.55–0.80), respectively.

The site-level models showed different patterns across rounds. The BMC model, trained further on local BMC data after initialization from the first round’s aggregated model, achieved AUCs of 0.69 (95% CI: 0.55–0.81) in both the second and third rounds, compared with 0.70 for the BMC-only baseline. The VA model, similarly trained further on local VA data, showed BMC test set AUCs of 0.68 (95% CI: 0.54–0.80) in both the second and third rounds. Across all rounds, AUCs on the BMC test set were comparable for the BMC, VA, and federated models (0.68–0.70). Confidence intervals were wide, reflecting the limited size of the BMC test set, and no pairwise difference between models were statistically significant.

At a fixed threshold of 0.5, the Round 3 federated model’s sensitivity was 16.0% and specificity was 96.3% on the BMC test set; at the training-derived Youden threshold (0.168), sensitivity was 64% and specificity was 62.5%, with corresponding positive and negative predictive values of 34.8% and 84.7%. The precision-recall AUC was 0.41. In a paired bootstrap comparison on the same test set, the AUC difference between the Round 1 federated model and BMC-only local model was −0.014 (95% CI, −0.123 to 0.099), and between the Round 1 federated and externally validated VA-only models was 0.005 (95% CI, −0.001 to 0.012)—neither difference was statistically significant at the 5% level. Comparisons in Round 3 against each site’s locally fine-tuned models were likewise not significant (versus the BMC model, −0.008, 95% CI, −0.053 to 0.036; versus the VA model 0.003, 95% CI, −0.002 to 0.008).

On the VA test set (n = 1955), the Round 3 federated model achieved an AUC of 0.70 (95% CI, 0.67–0.72), comparable to the VA local model (0.70; 95% CI, 0.67–0.72). The paired AUC difference between the federated and VA local models was below 0.001 in every round. It was statistically detectable in Rounds 1 and 2 given the size of the VA test set but of negligible magnitude (Round 1: 0.0006, 95% CI, 0.0001 to 0.0012). At the training-derived Youden-optimal threshold, sensitivity was 58.6%, specificity 72.2%, positive predictive value 46.0%, and negative predictive value 81.2%. The BMC-only model performed worse on VA data (Round 1 AUC 0.64, 95% CI, 0.61–0.67; paired difference from Round 1 federated model 0.056, 95% CI 0.030–0.082), whereas BMC models initialized from the federated model achieved VA test set AUCs of 0.69 and 0.70 in Rounds 2 and 3.

### 3.4. Calibration

**Figure 2:**
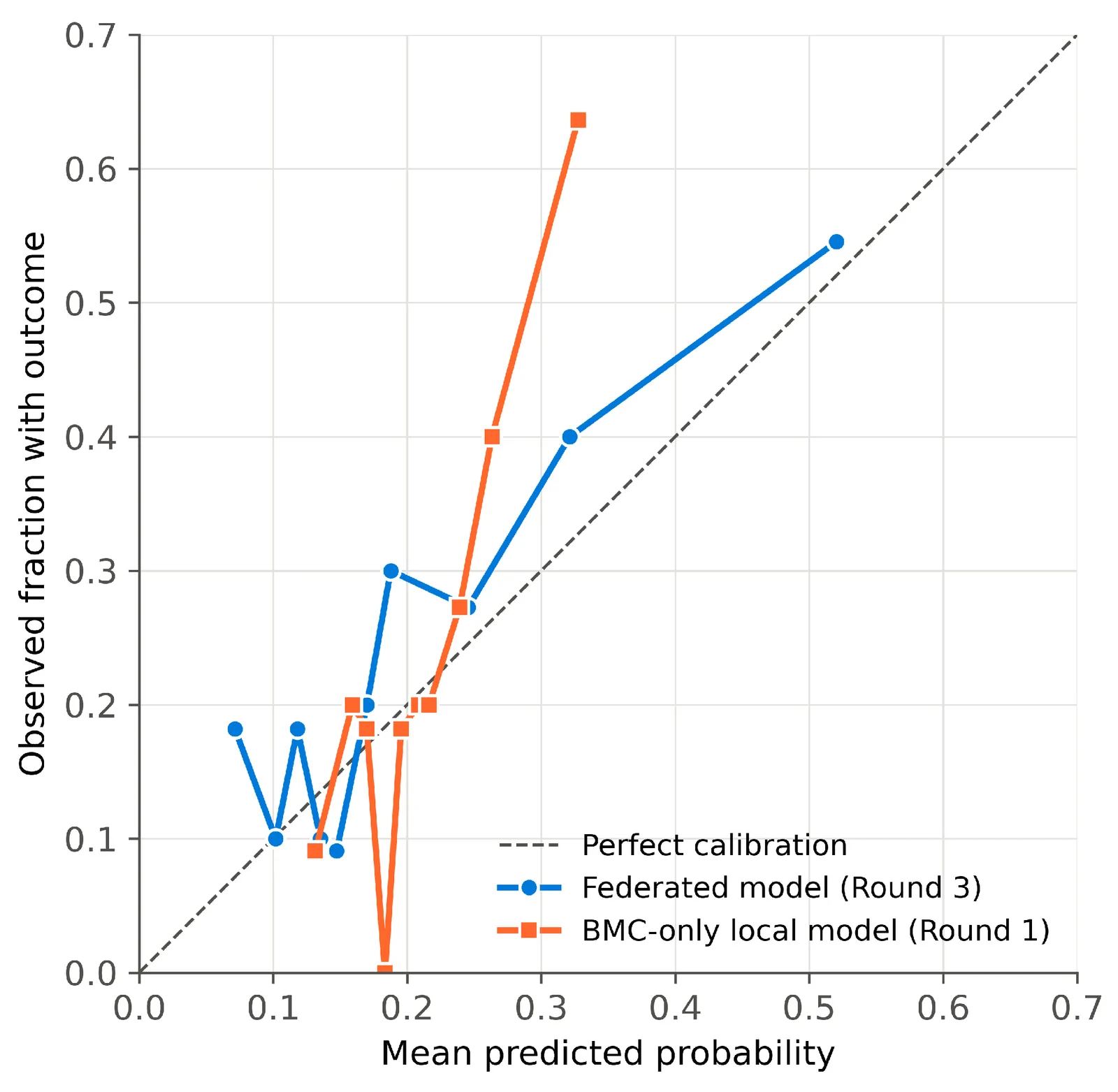
Calibration of the federated model (Round 3) and the BMC-only local model (Round 1) on the BMC test set. Points show the observed fraction with the outcome against the mean predicted probability within 10 quantile-based bins. The dashed line represents perfect calibration.

Calibration was assessed for the Round 3 federated model on the BMC test set. The calibration slope was 0.76, the calibration intercept was −0.09, and the Brier score was 0.171 (Figure 2). The BMC-only local model had a calibration slope of 2.30, an intercept of 1.82, and a similar Brier score (0.171), indicating predicted probabilities that were systematically too low and too compressed relative to observed outcome rates and federated model’s intercept close to zero. The federated model was well calibrated on the VA test set (calibration slope 0.93, intercept −0.03, Brier score 0.182).

### 3.5. Sensitivity Analyses

Under equal site weighting aggregation of the Round 1 models, which gave BMC 50% of the aggregation weight rather than approximately 5%, the federated model achieved an AUC of 0.70 (95% CI, 0.57–0.82) on the BMC test set, compared with 0.68 under the primary sample-size weighted scheme. The paired difference was 0.021 (95% CI, −0.016 to 0.062) and was not statistically significant. Calibration on the BMC test set was less favorable under equal weighting (slope 1.44, intercept 0.80 versus 0.83 and 0.02).

## 4. Discussion

We developed FedMann, a manually coordinated FL framework for fitting models across clinical sites and demonstrated its feasibility in a two-site application predicting non-receipt of guideline-concordant primary therapy in MM. Model performance was comparable between the federated model and each site’s own local model on that site’s test set (AUCs of 0.68 to 0.70), and the VA-only model showed no apparent loss of discrimination when externally validated at BMC. These results were obtained without exchanging patient-level data and with minimal infrastructure: after cloning the FedMann repository, each round required only five Python commands to execute, and two site representatives ran all three rounds in approximately 30 min. Site representatives executing the federated rounds require access to standardized, preprocessed data files (training and test sets), but data preprocessing and cleaning can be performed by data managers or informaticists at each site prior to framework execution, separating the technical requirements for FL coordination from the data engineering work.

Although demonstrated here using logistic regression, FedMann’s parameter-averaging approach is applicable to any model class for which weighted averaging of parameters is meaningful, including other generalized linear models and shallow neural networks. We hypothesize this manually coordinated workflow may scale well for models with modest parameter counts, where between-round verification of parameters by site representatives remains tractable. Tree-based ensembles fall outside the practical scope of this approach because their parameters are not directly averageable, and very deep networks would impose verification overhead inconsistent with the framework’s lightweight design. Within this scope, FedMann offers a practical alternative for groups that cannot deploy automated FL infrastructures such as VANTAGE6 [19], which generally require dedicated servers and firewall modifications and may be prohibitive for smaller institutions or consortia with limited IT resources or stringent information security requirements. While prior work has applied linear FL in oncology using automated infrastructures [11], FedMann extends this approach to settings where automated deployment is impractical. A centralized benchmark trained on pooled patient level data was not feasible in this application, as VA and BMC data-use agreements do not permit pooling patient-level records across sites. Our sample-size weighted aggregation follows established federated averaging conventions but has not been directly compared against such a benchmark.

The two participating sites differed substantially across all measured demographic characteristics (Table 1), most notably in sex distribution: the VA cohort was 96.3% male while the BMC cohort was 58.6% male. These differences are precisely the conditions under which simple external validation is most likely to fail, since a model trained predominantly on male VA patients may not transfer well to a more balanced population [20]. More explicitly, prior work has reported degraded performance when models are transferred to external sites [20], and we might therefore have expected the VA trained model to perform worse on BMC patients. Instead, performance was comparable across sites and model types. the VA-only model achieved an AUC of 0.68 on the BMC test set, compared with 0.70 for the BMC-only model, and the federated model achieved AUCs of 0.68 on the BMC test set and 0.70 on the VA test set, closely matching each site’s own local model (Table 3; Section 3.3). No pairwise differences on the BMC test set were statistically significant, although the wide confidence intervals mean that modest differences cannot be excluded. Federated learning is intended to let the smaller site’s data influence the combined model, rather than relying on a one-way transfer of weights from the larger site. In our application, however, sample-size-weighted aggregation gave BMC approximately 5% of the aggregation weight, so the federated model’s performance closely tracked that of the VA model and did not change across three rounds of aggregation. The clearest cross-site benefit was for the smaller site’s model: the BMC-only model, trained on 156 patients, performed worse on VA data (AUC 0.64) than the federated model (AUC 0.70), whereas BMC models initialized from the federated model reached AUCs of 0.69 to 0.70 on VA data. An equal-weighting sensitivity analysis, which gave BMC roughly ten times more weight, produced a slightly higher but not significantly different Round 1 AUC on the BMC test set (0.70 versus 0.68), so this weighting choice did not materially change our conclusions. Whether larger BMC cohorts would allow the smaller site to contribute more to the combined model remains to be established.

The absolute AUC values in this application were modest, and confidence intervals around site-specific estimates were wide, reflecting the limited size of the BMC test set and the substantial imbalance between the two sites’ sample sizes. No pairwise differences between models on the BMC test set were statistically significant. A single train/test split was used rather than repeated stratified resampling, since a gradient-based federated model does not map naturally onto conventional repeated cross-validation. These estimates and their bootstrap-based confidence intervals should be interpreted with that in mind. These results should be interpreted as feasibility evidence for the framework rather than as a clinically deployable model for predicting non-receipt of guideline-concordant primary MM therapy. Data heterogeneity also limited the number of predictors that could be harmonized across sites, which likely constrained absolute discrimination.

Privacy and security considerations warrant attention. Parameter exchange reduces the regulatory burden of moving patient-level data but does not, by itself, provide formal cryptographic privacy guarantees against attacks such as model inversion (reconstruction of training data from model parameters) or membership inference (determining whether a specific individual’s data was in the training set). To mitigate risks in this project, we limited sharing to model parameters and aggregate sample counts, used low-capacity linear models that have lower propensity to memorize individual examples, and executed transfers through an access-controlled, encrypted repository with logged events. Future deployments should consider integrating secure aggregation protocols and differential privacy mechanisms to provide formal guarantees while preserving model utility.

Future work on the FedMann framework should include evaluation across additional clinical sites, application to other model classes within the framework’s scope, and integration of secure aggregation and differential privacy protocols to strengthen formal privacy guarantees. Given FedMann’s reliance on git for parameter sharing and its flexibility to accommodate any model class trainable via gradient descent, scalability is primarily limited by model size rather than framework architecture. We expect that tens to hundreds of sites could feasibly participate with small-to-moderate capacity models using current resources. We hypothesize scaling to additional sites would likely improve model performance, especially when participating institutions contribute large-scale, complementary datasets with diverse patient populations and social determinants of health. However, this is not a scalability study. To leverage the full potential of federated data across heterogenous sites, future work should also explore representative learning and similar techniques that can better incorporate site-specific variables. These approaches could also address the challenge of handling missing input data by learning site-specific patterns of missingness rather than applying a uniform imputation strategy across institutions. The specific application presented here, a model predicting non-receipt of guideline-concordant primary MM therapy, would require additional development before clinical use, including expansion of the predictor set to capture social determinants of health and longitudinal utilization patterns, model calibration, and prospective evaluation of whether flagging at-risk patients for targeted interventions improves treatment receipt. A separate manuscript from our group is examining temporal trends in treatment receipt over the study period in more detail.

## 5. Conclusions

We developed and demonstrated FedMann, a manually coordinated federated learning framework that enables multi-institutional model fitting without exchanging patient-level data and without requiring dedicated FL infrastructure. Applied to prediction of non-receipt of guideline-concordant primary MM therapy across two sites with substantially different patient populations, a single federated model performed comparably to each site’s own local model on that site’s test set. Absolute discrimination was modest at both sites. Future work will evaluate the framework across additional clinical sites and model classes, examine alternative aggregation weighting, and integrate formal privacy-preserving protocols to support broader deployment.

## Figures and Tables

**Table 1:** Patient Characteristics by Site. Cells show the number (n) and percentage (%) of patients with each characteristic at each site. *p*-values compare characteristics between sites using chi-square tests of independence.

		Site				
		BMC (N = 261)		VA (N = 4887)		
Group	Category	n	%	n	%	*p*
Age Group (in years)	<55	42	16.1	320	6.5	<0.001
	[55–65]	84	32.2	1074	22.0	
	[65–75]	78	29.9	2189	44.8	
	[75–85]	46	17.6	1018	20.8	
	≥85	11	4.2	281	5.7	
	Missing/Unknown	0	0	5	0.1	
Sex	Female	107	41.0	176	3.6	<0.001
	Male	153	58.6	4706	96.3	
	Missing/Unknown	1	0.4	5	0.1	
Race/Ethnicity	Hispanic	47	18.0	243	5.0	<0.001
	Non-Hispanic Black	138	52.9	1525	31.2	
	Non-Hispanic White	58	22.2	2916	59.7	
	Other	9	3.4	152	3.1	
	Missing/Unknown	9	3.4	51	1.0	
Smoking Status	Current	23	8.8	1572	32.2	<0.001
	Former	85	32.6	2352	48.1	
	Never	151	57.9	827	16.9	
	Missing/Unknown	2	0.8	136	2.8	
Charlson Comorbidity Index (CCI)	0	78	29.9	744	15.2	<0.001
	1	114	43.7	2072	42.4	
	2	46	17.6	1632	33.4	
	3	1	0.4	364	7.4	
	Missing/Unknown	22	8.4	75	1.5	
Revised International Staging System (R-ISS) Stage	Stage 1	54	20.7	720	14.7	<0.001
	Stage 2	105	40.2	1416	29.0	
	Stage 3	56	21.5	1565	32.0	
	Missing/Unknown	46	17.6	1186	24.3	
Distance from Hospital (in km)	<25	194	74.3	1557	31.9	<0.001
	[25–50]	41	15.7	822	16.8	
	[50–75]	18	6.9	474	9.7	
	[75–100]	5	1.9	351	7.2	
	[100–125]	1	0.4	234	4.8	
	≥125	2	0.8	1100	22.5	
	Missing/Unknown	0	0	349	7.1	
Non-receipt of Treatment (Outcome)	No	201	77.0	3514	71.9	<0.001
	Yes	60	23.0	1373	28.1	

**Table 2:** Baseline Model Performance Across Sites without Federated Learning. The table compares discrimination performance of the BMC local and VA local logistic regression models across internal and external test sets, relative to where the models were trained.

Training Data	Testing Data	AUC [95% CI]
BMC	BMC	0.70 [0.56–0.81]
VA	VA	0.68 [0.54–0.80]
VA	BMC	0.70 [0.67–0.72]

**Table 3:** Model Performance on BMC Test Set Across Federated Rounds. Local: trained using only that site’s own data, with no cross-site parameter sharing. Locally fine-tuned: initialized from the previous round’s federated model and then trained further using only that site’s own data. Federated: produced by sample-seize weighted aggregation of that round’s uploaded site models. AUC = area under the receiver operating characteristic curve; CI = confidence interval.

Round	Site Model Type	BMC Local Model AUC [95% CI]	VA Local Model AUC [95% CI]	Federated Model AUC [95% CI]
1	Local	0.70 [0.56–0.81]	0.68 [0.54–0.80]	0.68 [0.55–0.80]
2	Locally fine-tuned	0.69 [0.55–0.81]	0.68 [0.54–0.80]	0.68 [0.54–0.80]
3	Locally fine-tuned	0.69 [0.55–0.81]	0.68 [0.54–0.80]	0.68 [0.55–0.80]

## Data Availability

Deidentified aggregated results are reported in the manuscript. Patient-level VA and BMC EHR data are subject to institutional data governance and cannot be publicly released; qualified researchers may request access through the respective institutional data access procedures. The FedMann codebase and scripts to reproduce analyses are available at: https://github.com/thunder001/FedMann (accessed on 23 September 2026).

## Supplementary Materials

The following supporting information can be downloaded at: https://www.mdpi.com/article/10.3390/jcm15218418/s1, Table S1: TRIPOD+AI Checklist.

## Abbreviations

The following abbreviations are used in this manuscript:

AUC	Area Under the Receiver Operating Characteristic Curve
BMC	Boston Medical Center
CCI	Charlson Comorbidity Index
CI	Confidence Interval
EHR	Electronic Health Record
FL	Federated Learning
IT	Information Technology
L-BFGS	Limited-memory Broyden–Fletcher–Goldfarb–Shanno
MICE	Multiple Imputation by Chained Equations
MM	Multiple Myeloma
NIH	National Institutes of Health
R-ISS	Revised International Staging System
TRIPOD+AI	Transparent Reporting of a multivariable prediction model for Individual Prognosis Or Diagnosis + Artificial Intelligence
VA	Veterans Affairs
VANTAGE6	An open source priVAcy preserviNg federaTed leArninG infrastructurE for Secure Insight eXchange
